# Adapting an adherence support workers intervention: engaging traditional healers as adherence partners for persons enrolled in HIV care and treatment in rural Mozambique

**DOI:** 10.1186/s13012-017-0582-z

**Published:** 2017-04-13

**Authors:** Carolyn M. Audet, José Salato, Sten H. Vermund, K. Rivet Amico

**Affiliations:** 1 Vanderbilt Institute for Global Health, Vanderbilt University Medical Center, Nashville, TN USA; 2 Departments of Health Policy, Vanderbilt University Medical Center, Nashville, TN USA; 3 Departments of Pediatrics, Vanderbilt University Medical Center, Nashville, TN USA; 4 Friends in Global Health, Quelimane, Mozambique; 5 Friends in Global Health, Maputo, Mozambique; 6 Department of Health Behavior and Health Education, School of Public Health, University of Michigan, Ann Arbor, MI USA

**Keywords:** Intervention adaptation, HIV/AIDS, Mozambique, Community-based support, HIV adherence

## Abstract

**Background:**

Systematic adaptation of evidence-informed interventions that increase retention in care and improve adherence to antiretroviral therapy (ART) are essential to ending the HIV epidemic in rural sub-Saharan Africa. We selected and adapted an adherence support worker intervention employed in Malawi for use by traditional healers in rural Mozambique. Given the levels of trust and dependence previously expressed by persons living with HIV (PLHIV) for traditional medicine, we adapted the program to engage traditional healers within the allopathic health system.

**Methods:**

Adaption followed a theoretically driven approach to intervention adaption: the Assessment-Decision-Administration-Production-Topical Experts-Integration-Training-Testing (ADAPT-ITT) model. Three rounds of performance feedback, based on theater presentations of the adapted intervention for stakeholders and idea generation, were completed with 12 groups from March to July 2016 to develop the final model. We offered healer support to 180 newly diagnosed HIV-infected patients.

**Results:**

Traditional healers were an acceptable group of community health workers to assist with patient adherence and retention. Traditional healers, clinicians, and interested community members suggested novel strategies to tailor the adherence support worker intervention, revealing a local culture of HIV denialism, aversion to the health system, and dislike of healthcare providers, as well as a preference for traditional treatments. Proposed changes to the intervention included modifications to the training language and topics, expanded community-based activities to support acceptability of an HIV diagnosis and to facilitate partner disclosure, and accompaniment to the health facility by healers to encourage delivery of respectful clinical care. PLHIV, healers, and clinicians deemed the intervention socially acceptable during focus groups. We subsequently recruited 180 newly diagnosed HIV-infected patients into the program: 170 (94%) accepted.

**Conclusions:**

Systematic translation of interventions, even between regions with similar social and economic environments, is an important first step to successful program implementation. Efforts previously limited to community health workers can be tailored for use by traditional healers—an underutilized and often maligned health workforce. It proved feasible to use theater-based performances to demonstrate delivery of the intervention in low-literacy populations, generating discussions about social norms, community concerns, and the merits of an acceptable strategy to improve retention and adherence to ART.

## Background

The long-term effects of delayed, interrupted, or discontinued antiretroviral therapy (ART) are severe for persons living with HIV (PLHIV), due to rapid disease progression and continued inflammatory-mediated aging processes [1, 2]. The risk of transmission to sexual partners increases with viral load, another incentive to treat early and effectively [3–7]. People with an especially low CD4+ cell count (e.g., <200 cells/μL) are particularly infectious and are themselves at enormous risk of lethal opportunistic infections and malignancies, most of which are preventable with successfully delivered ART [8, 9]. Effective treatment, however, relies not only on being prescribed ART but also on consistent, ongoing presentation at the care sites that re-issue and refill ART prescriptions, as well as daily adherence to medications.

In Mozambique, clinical services and medication for HIV are provided free of charge through National Health Service clinics funded in part by the President’s Emergency Plan for AIDS Relief (PEPFAR). Implementation of evidenced-based HIV care and treatment services is guided by the Centers for Disease Control and Prevention (CDC), with efforts to create sustainable, locally owned, and country-driven programs that fit within the context of a comprehensive health system [10, 11]. With a national influx of US$234 million in 2015, the proportion of eligible patients enrolled on ART has increased to 53% [12]. Despite these investments, estimates suggest that less than 50% of patients enrolled on ART remain in HIV care at 1 year after starting ART in rural Mozambique [13]. Failure to successfully adapt and/or implement evidence-based practices to the local cultural context may be, at least in part, to blame for the poor clinical outcomes observed [14].

To better understand reasons for discontinuing HIV care in Mozambique and identify avenues for intervention, a cohort of PLHIV who were lost to follow-up was established in the Zambézia province through tracing and in-community outreach [15]. The primary reasons cited for abandoning HIV care included concerns about disclosure/stigma [15], fear of gender-based violence [16], lack of food and/or transportation [15], health system quality [17], patient-provider conflict and distrust [15, 18], and a preference for use of traditional medicine [16, 19]. Recent joint efforts between Friends in Global Health (FGH)—the local non-governmental organization of Vanderbilt University Medical Center—and the provincial Ministry of Health to improve retention in HIV care involved the implementation of an expanded support system (peer system). The peer system incorporates expert peer navigators to assist PLHIV at the clinic, peer educators to educate PLHIV about HIV while waiting for clinical services and convince PLHIV who have abandoned care to return, “male champions” to counsel male partners and encourage them to get tested with their pregnant wives, and traditional birth attendants to promote HIV testing and treatment initiation [20]. Presently, the focus of intervention efforts using the peer system is on promoting initiation of HIV care and addressing neglected issues of stigma, food, transportation, and the potential for gender-based violence. However, little attention is focused on strategies to increase patient trust in allopathic diagnosis or treatment. Trust, defined as “the willingness of a party to be vulnerable to the actions of another party based on the expectation that the other will perform a particular action important to the trustor, irrespective of the ability to monitor or control that other party” [21], is central to the patient-provider relationship, likely mediating patient uptake of medication, adherence, and retention [22]. The most important factors influencing patient trust include patient perception of physician concern, compassion and caring for their well-being, and the willingness of the physician to advocate for their patients interests [23, 24].

In Mozambique, PLHIV who initiate care and fail to return receive at least one home visit from a peer educator, but little ongoing support is provided. In-community services provided by established, respected community members offer high feasibility, have embedded cultural relevance, and can produce sustainable strategies to support PLHIV. An untapped resource for such programs is traditional healers.

To explore a new model of treatment partnerships, based on the effective strategies of responsible task shifting [25] and expert peer support [26], we partnered with traditional healers in Zambézia. Healers are a well-respected and culturally acceptable alternative to allopathic care in much of sub-Saharan Africa, including Mozambique. The majority of traditional healers belong to the national organization of AMETRAMO (Associação dos Médicos Tradicionais de Moçambique [Traditional Healers Association of Mozambique]). In 2015, the local Namacurra chapter of this association approached FGH to request a formal role in assisting PLHIV. The role of these healers in promoting HIV care utilization can be negative (promoting delays and discontinuation of care [27, 28]) or positive (strong advocates for patient health [29, 30]). A lack of HIV/ART knowledge, positive beliefs about HIV care, and skills to motivate individuals to access and remain in HIV care limit the potential positive influence of traditional healers [31–35]. Thus, we believed the development of a program focusing on the delivery of quality HIV care promotion, education, and counseling from in-community traditional healers required careful efforts to build trust and strong working relationships between care sites and traditional healers, provide HIV treatment and counseling training for healers, and establish clear guidelines as to the roles healers would play in the treatment of PLHIV. Therefore, our goal was to systematically adapt a community health worker adherence support worker intervention for use by traditional healers in Zambézia, with the idea that programs providing HIV care and treatment services in a similar context (sub-Saharan Africa, preference for traditional medicine, poverty, low levels of education) could tailor our final intervention model for local use. We sought to answer three questions: (1) Can traditional healers be trusted by community members and health care providers to deliver HIV care and treatment support? (2) What types of support are acceptable for them to provide? (3) What is an effective approach to deliver this support?

The Assessment-Decision-Administration-Production-Topical Experts-Integration-Training-Testing (ADAPT-ITT) model provides a systematic framework to gain feedback and pilot a chosen behavioral intervention with key stakeholders [36]. Methods used in ADAPT-ITT include formative research to identify evidence-based interventions for consideration, selecting the appropriate intervention for adaptation, working with an expert panel to make preliminary adaptions, and presenting the adapted intervention to stakeholders in a theater performance format to elicit feedback for intervention improvement. In addition to creating a localized version of an evidence-based intervention, involving those who will implement and benefit from the intervention increases intervention fidelity and effectiveness [14]. We describe herein the process of employing the ADAPT-ITT model (specifically the steps of Adaption, Production, and Topical Experts) to adapt our evidence-informed intervention while maintaining the underlying behavioral model. Acceptability, as well as patient and healer preferences for support approach and delivery, was assessed through participant reactions to theater performance and through testing uptake of this service by newly diagnosed HIV patients.

## Methods

We began by gathering information about similar interventions employing non-healers in nearby countries [37–44] and through interviews and informal discussions with healers, people enrolled in HIV care and treatment, and clinicians living in the target community. An evidence-informed intervention was considered relevant if it was designed for PLHIV with low-literacy levels that lived in rural areas with poor health service coverage. We ranked the interventions based on the inclusion of four elements: (1) theory-driven; (2) included training on counseling PLHIV and their families; (3) prior implementation in rural Africa; and (4) inclusion of a component addressing stigma. Using these criteria, we chose the Adherence Support Workers program [38], developed by FHI 360, to provide an optimal foundation for our intervention. The Adherence Support Workers program includes three primary activities: (1) education and psychosocial support to PLHIV initiating/continuing ART; (2) referrals to specialized clinics as needed; and (3) participation of support workers as members of the ART clinical team [38]. This program had been previously implemented in rural sub-Saharan Africa, was socially acceptable, and provided accessible education and training manuals [45].

### The adaption process

The Adherence Support Workers program provided an appropriate backbone for our intervention to engage traditional healers as treatment partners; however, we needed to adapt the program to fit a different type of support worker (a traditional healer with low-literacy levels), support PLHIV living in a unique cultural region (rural central Mozambique), and be written in both Portuguese and Echuabo (the predominant local language). Thus, we employed the framework below to better understand the acceptability of the program, including the necessary skill development to ensure healers and clinicians could partner to improve adherence to ART and retention in care.

### Theoretical framework

We used the situated-Information Motivation Behavioral Skills model of Health Care Initiation and Maintenance (sIMB-CIM) to guide our focus group interview questions and analysis in efforts to adapt our chosen intervention [46, 47]. The sIMB-CIM adopts the core determinants of health behavior identified in the IMB model [48–50]. In the sIMB-CIM, the specific content within the core IMB areas (information, motivation, and behavioral skills) is tailored to the process of initiating and sustaining an established behavior over time, with an emphasis on situating content to the cognitive-affective social-cultural environment in which care is negotiated [46]. This model is particularly well-suited to Mozambique’s collectivist culture, in which people tend to view themselves as members of groups (family units, linguistic groups) and typically consider the group’s needs over their own individual needs [51]. Given the local experience of the intervention adaptation team, specifically working with Mozambican traditional healers, we were able to identify potential strengths and weaknesses in each of the core areas. During the Administration-Production-Topical Experts-Integration process, we focused on three things: (1) facilitators and barriers to acceptability of healers as treatment partners; (2) strategies to encourage specific actions necessary to improve health outcomes (e.g., healer advocacy for patient rights if clinicians are not providing satisfactory care); and (3) identification of skills needed to carry out these actions (e.g., ability of healers to provide safe and effective counseling for partner disclosure of HIV status).

### Theater presentations

Three theater presentations were developed in association with members from AMETRAMO, PLHIV in Namacurra, clinicians providing HIV care and treatment, FGH community health workers, two local theater groups, and researchers from Vanderbilt University Medical Center. This development process included discussions regarding the current retention and adherence problems among PLHIV, specific challenges patients encountered at the health facility, with a traditional healer, and/or at home, and the contributions of community health workers in the region. After the first presentation was performed five times (to stakeholder groups in no particular order), we met to discuss issues that were raised by participants and to create the subsequent theater presentation. The second and third presentations were made increasingly specific in efforts to address participant feedback and concerns (e.g., how exactly would a person select a healer? What happens if your healer demands money?). The use of theater presentations as a communication tool was acceptable and welcomed by community members who had limited ability to read and write. The three theater presentations that were developed, modified, and shown are as follows:

- *Theater presentation 1*: In rural Mozambique, a woman begins to complain of headaches, body aches, and fever to her partner. The partner defies local stereotypes, accompanying his wife to a traditional healer and, subsequently, the health facility for testing and treatment. While at the health facility, the nurse refuses to see the patient because of her late arrival. One week later, the healer checks on his patient and finds her situation has worsened. He accompanies both partners to the health facility, where the woman is finally diagnosed with HIV and initiated on treatment. When the woman returns home, she refuses to take the medicine, instead asking her husband to find her some traditional herbs in the forest. Her healer finds her throwing the pills away. He teaches her about HIV and the importance of adherence and explains the types of side effects the drugs could cause. She recommits to taking her medication.
- *Theater presentation 2*: A female patient arrives at the health facility for a consultation. After receiving an HIV diagnosis, she is asked if she would like to have a traditional healer as a treatment partner. She is shown a poster with dozens of photos and information about local healers, and she chooses one that lives in her community. Fearful of the consequences of her diagnosis, she returns home and says nothing to her husband. Later that week, her treatment partner comes to visit. She explains her fear of disclosure and her misunderstandings about HIV and HIV treatment. The healer (male or female) provides counseling and, after obtaining her approval, counsels the couple together about her HIV status and the desirability for the husband to be tested for HIV.
- *Theater presentation 3*: A healer discusses the typical side effects of ART medication with his patient, explaining that this patient’s symptoms are serious enough to warrant a facility visit. The patient is too sick to walk to the hospital, so the healer provides transport on the back of his bike for the one hour ride. When they arrive at the health facility, the healer asks the patient for some money to buy water and bread. The patient now has to decide: does he/she argue with the healer or pay him/her?

### Focus group methodology

We conducted 12 focus group discussions (FGD) following the theater presentations with the following groups of people: PLHIV, defined as HIV-infected individuals enrolled in care (4 FGD; *n* = 33 total); community members of unknown HIV status (3 FGD; *n* = 30 total); healers practicing in Namacurra (3 FGD; *n* = 28 total); and clinicians providing HIV care and treatment in Namacurra (2 FGD; *n* = 17 total). PLHIV were identified while waiting in line for HIV services on the morning of the focus group; 35 were approached and 33 agreed to participate. Two community leaders traveled to each of three neighborhoods to spread the word about the theater presentation. Of those community members who attended the presentation, we intentionally selected participants to create groups of diverse age and gender. Healers were selected from the same three communities as participants. Each healer had to be living in the respective neighborhood, involved in actively treating PLHIV, and interested in learning more about the program. All healers from each neighborhood who were interested were allowed to participate. Participants were all 18 years of age or older and lived in the catchment area of the Namacurra health facility.

#### Procedure

Participants in all focus groups watched a theater presentation of the intervention; healers, PLHIV, and community members were provided the presentation in the local language (Echuabo) while clinicians preferred to hear it in Portuguese. Before the presentation, we explained the purpose of the study, namely, that we were going to model a possible intervention in which healers provided HIV treatment support for PLHIV. Between each group of sessions, we revised the theater presentation to accommodate suggestions made by the previous groups. Open-ended, semi-structured questions were focused on current barriers to HIV treatment retention and adherence, ways in which healers could assist PLHIV in overcoming these barriers, and the challenges of having a healer as a treatment partner. Among healers, additional questions about compensation, the potential for mixing traditional and allopathic medication, and their needs for training were also discussed. All focus groups were audio recorded and transcribed into Echuabo (PLHIV, healers, community members) or Portuguese (clinicians) and subsequently translated into Portuguese (if necessary) and English. Portuguese and English translations were done separately by two individuals to ensure accuracy. One person conducted all FGD sessions with the assistance of two trained researchers. Translations were completed by qualified Mozambican translators living in Zambézia province.

### Data analyses

Framework analysis was used to identify main themes from our FGDs (Table 1) about the drivers, core facilitators, and barriers to acceptability of the traditional healer-based intervention approach. Four code maps were developed to categorize data: (1) social, structural, and informational drivers, facilitators, and barriers to acceptability of healers as adherence partners; (2) educational and counseling strategies that healers could use to encourage retention in care and adherence to ART (e.g., partner counseling); (3) the role that healers could play in ensuring that respectful care is provided by clinicians; and (4) recommendations for the development of a “best practice” intervention strategy, including the necessary information, motivation (for healers and PLHIV), and appropriate behavioral considerations healers would be required to follow. Data analysis was conducted using MAXQDA 12 software (VERBI GmbH; Berlin, Germany).

**Table 1:** Participant questions

Theater presentation 1
1. What are some factors that stopped the patient from adhering to his medication?
2. What could a traditional healer do to help the patient with those problems?
3. What are some other factors that stopped the patient from returning to the health unit for the consultation or to collect her medicine?
4. What could the healer have done to help the patient with that?
5. What was your reaction when seeing a traditional healer providing support to an HIV positive patient?
6. How did the healer provide support to the patient?
7. What did you like the most about the support they gave?
8. What did you like the least about the support they gave?
9. How can we improve the way in which the healer provided support?
10. What are some barriers to having a traditional healer as a support worker?
Theater presentation 2
1. In this theater presentation, our patient decides to work with a traditional healer as her treatment partner. But, she could have also decided not to accept. What do you think are some of the reasons she should say yes?
2. What are some of the reasons she should say no?
3. If we employed healers as treatment supporters, how should we choose which healers to work with?
4. In our presentation, the patient says yes to working with a healer. If you were in her place, how would you decide which healer you would work with? What information would you like us to provide to help you make that decision?
5. What is the best way to educate a patient about the different healers he/she can work with? Should the nurse provide a list of possible healers? What is your idea?
6. Once the patient has picked a healer, what is the best way of connecting them?
7. How often should the healer visit the patient?
8. Should healers be paid for their work?
Theater presentation 3
1. Are you worried that a healer support worker will ask you for additional money?
2. [Healers] Describe the incentive that would allow you to do this work without asking a patient to pay additional money.
3.What is a reasonable incentive to provide a healer?
4. What is the motivation that drives traditional healers to help a patient living with HIV?
5. What is the best way to ensure a healer is doing a good job? How can a patient complain if the healer is not working well? What can the healer do if the patient refuses their assistance?

### Acceptance of the intervention

From March to August 2016, a study coordinator recruited 180 prospective participants into the study on the same day they received an HIV diagnosis from the Volunteer Counseling and Testing service at the primary hospital in Namacurra district. Enrollment in the healer adherence support program was recorded to determine program acceptance.

## Results

### Adaptation process

One hundred eight people watched one of the three theater presentations and participated in FGDs (Table 2: Participant characteristics). Differences and similarities between the original adherence support worker intervention and the proposed adaptation of the traditional healer partner intervention are summarized in Table 3. In the proposed intervention, healers would provide three distinct types of support to PLHIV: (1) education and psychosocial support to PLHIV initiating ART; (2) accompaniment to health facility to advocate for patient health; and (3) participation as members of the ART clinic team to ensure seamless care at the clinic and in the community. Main content themes emerging from discourse focused on acceptability of the intervention, as well as reactions to proposed education, advocacy, and counseling strategies specific to the participant-traditional healer dyad and the participant-healer-clinician triad, are summarized below. These were used to create the adapted intervention approach.

**Table 2:** Study participant characteristics

Study characteristics	PLHIV	Community members	Traditional healers	Clinicians	Total
Total	33	30	28	17	108
Presentation 1	2	1	1	1	5
Presentation 2	1	1	1	1	4
Presentation 3	1	1	1	0	3
Median age in years (IQR)	38 (29, 48)	39 (23, 56)	50 (44, 63)	32 (28, 36)	40 (28, 51)
Number of women (%)	17 (52%)	25 (84%)	18 (62%)	11 (65%)	71 (66%)

**Table 3:** Original adherence support worker responsibilities compared to the modified role for adherence support provided by traditional healers, Zambézia province, Mozambique

Original adherence support worker responsibilities	Modified traditional healer responsibilities
1. Provide education and psychosocial support to PLHIV initiating/continuing antiretroviral therapy (ART).	1. Provide education and psychosocial support to PLHIV initiating ART.
a. Support PLHIV to adhere to clinical monitoring protocol (retention in care). b. Support PLHIV to adhere to medication.	a. Support PLHIV to adhere to clinical protocol (retention in care). b. Support PLHIV to adhere to medication. c. Provide couples-counseling to support safe partner disclosure. d. Provide education on nutrition needs to limit the impact of drug side-effects. e. Assure PLHIV that illness is not due to witchcraft.
2. Provide referrals to specialized clinics, as needed.	2a. Provide accompaniment and referrals to health facility as needed 2b. Perform advocacy role for patient health at health facility. 2c. Report poor treatment of PLHIV to facility director.
3. Participate as members of ART clinic team.	3. Participate as members of the ART clinic team.

### Can healers be trusted to deliver HIV care and treatment support?

Clinicians, PLHIV, community members, and healers themselves expressed varying degrees of confidence in the idea of healers acting as adherence coaches for ART. The most confident were clinicians, who found themselves overwhelmed with patients needing adherence support. Clinicians acknowledged their inability to provide community-based support, with 12-month retention below 50%; healers were therefore seen by clinicians as an alternative avenue to gaining patient confidence.

Everyone needs, and the healer generally here in Africa, in our country in particular, is the first person who we trust, since our traditions there’s no one that has surpassed the healer. (physician, man, 43 years).

Community members also saw benefit to this collaboration:

Practically the healer is the person from our community whilst the nurse is the person that stays a bit distant from us, at the hospital. Then it is very good that [the healers] are the ones that facilitate our process … When he sees that this paracetamol is not giving the [desired] effect, he takes you to the hospital to go seek other medication. (community member, man, 68 years).

Healers themselves were enthusiastic about the potential collaboration, with a few describing their current role as informal treatment partners. None of the healers had previously been formally trained to assist PLHIV with medication adherence; they were improvising. They highlighted their role in the process by referring to assistance in medication pickup, pill counting, nutrition, and even pressuring PLHIV to take their mediations.

My friend you cannot play with this medicine because I suffered a lot by accompanying you to the hospital. So you cannot bring the medicine and keep it here [not take it]. You have to take it to get better. (traditional healer, woman, 45 years).

Several concerns about the creation of this new role were voiced by PLHIV and community members. Five of the PLHIV were angry at healers for delaying their access to HIV care while taking their money and flatly refused to work with any healer. Others were open to the idea, but were fearful of intentional or unintentional disclosure of the patient’s status.

I liked it, but I just want to ask, I don’t know those healers, when that time comes, you will talk to them for them to be people who keep secrets? If he sees a patient or collects medicine, or is sought after by a patient and he accompanies him to the hospital, will he keep it in his heart? Will he keep the secret to himself? (patient, woman, unknown age).

PLHIV were concerned about how healers would be compensated for their role. This area was intentionally left vague during the presentations to elicit participant feedback on the most acceptable method of fairly remunerating a treatment partner. PLHIV feared that uncompensated healers would expect PLHIV to pay them for their assistance, which was deemed unacceptable.

What will the healer’s remuneration be? Will it be that the patient is the one that’s going to pay or the health [wellbeing] of the patient is what’s going to pay the healer? I say this because you could have a case one day at dawn I need help. I go all the way to the healer’s house and find him sleeping, and in turn he could say that ‘I can accompany you to the hospital, but you will give me some money’. And it’s that that we’re afraid of. (community member, man, unknown age).

Healers uniformly supported the intervention, but were split on the issue of payment. Some found the intervention acceptable without payment; others suggested the position required too much work to be unfunded.

… but we carry the sick person and bring them to the hospital, and we work without them giving us anything. We forego going to the fields (to work the land); we don’t attend our other patients, in order to accompany the one who needs the hospital. That is work, and ultimately you leave us with nothing. (traditional healer, woman, 40 years).

While some healers claimed that all they wanted was “for people to be well in the community” (traditional healer, woman, 56 years), the program was ultimately more acceptable if PLHIV felt unpressured to provide compensation.

### Acceptable mechanisms of support provision

#### Reassurance in their allopathic diagnosis and treatment

A history of distrust and dislike between patients and clinicians has led PLHIV to be skeptical of any diagnosis or treatment provided at the health facility. Healers have played into this dynamic by providing alternative diagnoses that are more socially acceptable, better to have been cursed by your mother than to have AIDS. One woman nervously stated, “Patients say this disease does not exist, we are being lied to” (patient, 45 years). Worse, rumors that ART is the cause of HIV suggest a community-level fear that clinicians will intentionally harm them. Another woman explained, “There in the bush [community], they say that those pills are giving us the disease” (patient, man, 40 years). PLHIV who feel that clinicians are lying to them about their HIV diagnosis or who are trying to infect them with the virus through medication are likely to have lower retention and adherence outcomes among their patients.

Healers have one important advantage over clinicians and counselors: people believe the healer’s diagnosis more readily than that of an allopathic provider. A patient explained:

It would be good if the healer said, ‘There is no spell [curse], I just want to accompany you to the hospital, for you to go together, accompany you, make you go through all the places you need to. If I leave you alone, you will not get there.’ (patient, woman, 32 years).

Healers informally provide retention support to PLHIV by convincing them that their HIV diagnosis is valid and that the medication will allow them to rebuild their health. One healer described the process of gaining patient trust:

I have a patient in Natete, I treated him and then I accompanied him to the hospital. I was given medicine, but he didn’t take it. When I asked he said, ‘Those chemicals they gave me, they say it’s called AIDS. So I cannot take them because what I have is not AIDS.’ I tried to make him aware, I even brought him here again … Today when I passed by he said he is a little better. (traditional healer, woman, 45 years).

Healers have to cultivate a relationship with their PLHIV to overcome widespread distrust of the health system. Strategies to create trust include frequent social visits, accompaniment to the health facility, and home-based counseling. One healer explained:

If you find a patient who does not want to go to the hospital, you have to go up to him and try to raise his awareness, tell him, ‘You won’t go on your own; I will accompany you. If we have to wait for a long time before we are seen I will be there with you.’ (traditional healer, woman, 45 years).

#### What types of patient support are acceptable?

##### Patient-healer dyad interaction

Participants described value in healers providing medication pickup if the patient is too weak to travel, as well as home-based counseling about HIV adherence, nutrition, and relationships.

I had my patient that I brought and was given medicine. When they gave it to him, he took one, two and stopped. When I checked I found all the pills were dirty [thrown in the dirt], and I said, ‘Wow, can you take more this way?’ She left them in the ashes, so I asked her to give me the prescriptions, since I didn’t have transport to take her, and she would get drowsy. So I went to get other medicine. She started to take them and finished them all. Yesterday when I went to see the pills, there are leftovers, and I advised her that while she takes the medicine she must feed herself well, eat greens from cassava, sweet potatoes, peanut, and to eat small things, to eat at least three times a day; but now she is getting better. (traditional healer, woman, 45 years).

All groups recognized the importance of family in ensuring engagement in care and retention to medication, as well as the acceptance of healers acting as adherence supporters. Revealing a positive HIV status to a partner can result in divorce. Our theater presentation included a scene in which the healer counsels the husband of a recently diagnosed HIV-infected woman. All groups agreed that “if the wife does not have her own husband’s support, who she lives with, I think that the possibility of continuing the treatment is much smaller” (clinician, woman, 27 years). PLHIV with supportive families employed family members to fetch medications, help them prepare foods, and give reminders to take medication. One woman expressed her surprise at seeing the husband provide support for his wife in the theater presentation, even after her diagnosis. She said:

I like the part of the husband the most, when he understood about the disease, because there are some, some that when they find out that the wife is infected, from there he would only be rejecting her because [he wonders] where did you bring this disease from? Since he was tested and he didn’t have it, he would be rejecting her. He would not [normally] support her, but he supported her, and he even continued taking care of his wife. That is what I liked the most. (community member, woman, 73 years old).

##### Patient-healer-clinician triad

In addition to community-based education and counseling services for the patient and family, healers can also provide clinic-based advocacy for PLHIV living in Namacurra. There were two primary roles participants suggested. First, they underscored the need for clarification of any instructions and explanations provided by clinicians, with translation to the local language when necessary. Second, they advocated a defense of patients’ rights if poor treatment was witnessed.

Clinical providers may make every effort to deliver quality care, but PLHIV with low levels of education and little exposure to the germ theory of disease may have difficulty grasping certain concepts, including “virus,” “viral load,” “CD4 count,” and the need to adhere to medication forever. One woman explained:

I want to talk more about the nurse. That time he informed [me] about the disease and the condom. He should have explained more, right? Take that condom, because it is the worst example. In those health centers where many [people] don’t understand that thing of condoms [what condoms are], luckily, even in the play, that patient asked, ‘What is that? Is it a sweet?’ I don’t know … [paused as she was thinking]; he [the clinician] could open it, if there were that piece of wood [note: wooden penis dummy] there to be able to explain more how it is used, yes! And about the disease he has to explain what HIV/AIDS means very well. (community member, woman, 73 years old).

Healers can play a role by translating details of the illness and treatment to PLHIV. They have more time with the patient and understanding of local illness narratives to effectively convey information to PLHIV. In addition to providing multiple forms of translation, healers can also act as patient advocates. Community members complain of clinicians refusing to provide treatment, long wait times, and inaccurate diagnosis/treatment delivery. A woman reported, “Sometimes you go there very sick; you find the nurse talking on the phone. He does not even receive you;. he is more concerned about the phone; and you, the patient, he ignores you” (community member, woman, 19 years). A healer present at this encounter can both document this behavior and ensure that the patient is seen in a timely manner.

### Final healer support model

Discussion with focus group participants led to the creation of a final version of the model, including selection of healers, training needs, socially acceptable assistance strategies, payment options, and the creation of a system to identify poorly performing healers. PLHIV were entirely focused on the selection of a healer who would maintain their confidentiality; healthcare workers focused their concerns on a healer’s ability to learn counseling skills and the technical aspects of HIV care. These concerns supported the use of the technical training and exams developed by the Adherence Support Worker to ensure a healer has the competence to complete his/her job. PLHIV preferred the option to select their own healer from a book of images (including a map to show where the healer lived). Some PLHIV were adamant they wanted a healer who lived far away, while others wanted someone in the same neighborhood. Concerns about an opposite-gender healer providing care supported ensuring same-gender supporters would be available to intervention participants.

Healers, PLHIV, and community members agreed that home visits every two weeks for the first few months of treatment would be acceptable. During one of these visits, the patient’s partner would be counseled (if the patient agreed) and would be accompanied to the health facility for testing. During each visit, the healer would count pills, ask about side effects, and talk with the patient about nutrition. One clinician suggested:

We also have to include in the demonstration of enriched porridge, key messages for cooking and nutrition because there they only talk about leaves, they don’t talk about moringa [scientific name: Moringa oleifera]. So he has to have basic knowledge to be able to help the patient. They cannot have the theory only; they also need the practice, for them to have a day where they can demonstrate the foods. It may not necessarily be the porridge, it could be cassava… (clinician, woman, 30 years).

Healers would accompany their patient for the first three clinical visits, all of which should occur in the first month after diagnosis, and subsequently for follow-up every three months. PLHIV were concerned that healers would be spending a great deal of time assisting them, with little financial incentive. Healers requested rubber gloves (to protect themselves from patient blood during traditional treatments) and food, as well as hats and t-shirts to identify their position. They understood the restriction in funding as established by Ministry of Health policy. Given their requests, healers selected as a treatment partners in the Zambézia program receive a branded hat and t-shirt, rubber gloves, and additional incentives, including food, based on the number of PLHIV they supported. Given concerns expressed both by PLHIV and healers, the maximum number of PLHIV was three per healer.

PLHIV were also concerned about the consequences healers would face if they disclosed a patient’s HIV status or otherwise abused the relationship. As requested, a full-time coordinator was placed at the study site and his/her phone number was distributed to all PLHIV in case of problems. Healers were censured by the Chief Medical Officer at the site and removed from the program if problems were reported.

### Uptake of the intervention

FGH assessed participant uptake of the final intervention model by describing each aspect of the program to newly diagnosed PLHIV, via a study coordinator, after they completed standard counseling and testing. Of the 180 newly diagnosed PLHIV approached to participate in the program, 170 indicated their preference to have a healer act as an adherence partner vs. standard of care (SOC) (94%). No compensation was offered to these participants. Those who refused cited the dislike and/or distrust of traditional healers. Of those who accepted, 93 (55%) were women and 77 (45%) were men. The median age of participants was 30 (interquartile range (IQR) 23–39). Among participants, 115 (68%) were married/in common law relationships, 30 (18%) were single, 17 (10%) widowed, 6 (4%) divorced, and 2 (1%) were separated from their partner. Of the 114 participants with an active sexual partner, 65 (57%) of the partners had an unknown HIV status, 8 (7%) recently tested HIV-negative, and 41 (36%) were HIV-infected.

## Discussion

We documented our systematic adaption of the Adherence Support Worker [38] program for the incorporation of traditional healers as adherence partners in Zambézia province, Mozambique. Our study highlights the acceptability of engaging traditional healers as potential health care extenders, describes the social conditions under which healers could prove effective allies, and provides detailed direction on how best to engage healers to assist patients. Given the large numbers of healers in rural regions [27, 52–60], our findings are likely to be relevant for other HIV care and treatment programs in sub-Saharan Africa. With pressure to scale up HIV care and treatment programs, patients are discontinuing treatment at an unacceptable rate [61]. While projects have engaged healers in efforts to refer patients [58], provide counseling [29, 62], and disseminate HIV prevention information to community members [63], there is not an established and effective community health worker intervention tailored for healer use. Our adaptation can be the blueprint for such a program throughout sub-Saharan Africa (SSA).

Our adaptation was deemed acceptable and feasible by clinicians, healers, community members, and the majority of PLHIV during focus group discussions. Rollout of the newly adapted study indicated high acceptance among newly diagnosed HIV-infected patients. In communities where healers are trusted health providers, healer participation in the health system may lead to improvements in health provider trust and belief in allopathic diagnosis and treatment. Versions of an adherence support partnership have been implemented in many contexts, including Haiti [44, 64], Rwanda [65], Uganda [66], and South Africa [40, 67–69], and the results of these programs have been positive. In Mozambique, community health workers do at least a single home visit after diagnosis, in addition to searching for patients who are lost to follow-up, but there is no consistency in the person providing support. Poor outcomes may be linked to this lack of provider consistency and limited relationship-building that occurs in this system [70–72]. With the engagement of a single healer-patient dyad, our intervention allows healers and patients to develop a stronger relationship, given repeated contact and tailored support services.

Healers, patients, and clinicians reached agreement regarding the types of support healers could provide. Primary changes in the cultural adaptation to the Adherence Support Worker for use by traditional healers included translation of training materials into Portuguese and Echuabo, the selection of traditional healers as adherence partners, a patient’s ability to select the healer of his/her choice (and “fire” him/her if necessary), the addition of non-monetary incentives for the adherence partners, partner accompaniment to clinical visits, the role of healers as patient advocates in situations of poor clinical service delivery, and the role of healers in assuring their patients that their diagnosis is real and not the result of a curse. Healers can be chosen by newly diagnosed HIV-infected patients through a Facebook-style picture book, organized by healer home location, so people can choose a healer close or far away, as per their preference.

While the skills and challenges in providing ART adherence support were similar to those described in the Adherence Support Worker manual, our Mozambique population requires unique support to facilitate HIV care. Primarily, the lack of trust that exists between the health facility and the community presents a challenge to retention and adherence (both for HIV and for any chronic disease). Currently, PLHIV are comfortable challenging their HIV diagnosis; the traditional illness known as matoa (local illness with similar symptoms to HIV) is more socially acceptable [58, 73, 74]. With trained healers reaffirming their HIV diagnosis and the value of ART medication, we anticipate PLHIV will accept their diagnoses and treatment plan more readily. A previous study found seriously ill patients routinely assessed clinician trustworthiness against their own expectations [23]. Given vast cultural differences that divide clinicians from their patients in rural Mozambique, a cultural broker, like a trained traditional healer, may be necessary to bridge the gap. Among PLHIV who do not value traditional medicine, we expect many to refuse this support. However, we also predict those PLHIV who refuse may also be the ones who least need assistance in adhering to care.

As an adaptation study, our study has methodological limitations. First, we sought to adapt a version of a previously successful intervention, not test its efficacy at improving retention in care or adherence to medication. This will be determined by an ongoing study that we are currently conducting. More must be learned to predict which healers will prove the best coaches for specific PLHIV. Hence, we will collect data on healer personality, empathy, emotional intelligence, perspective taking, and social reasoning, along with data on patient personality, social reasoning, and HIV stigma, to determine the best system for matching provider and patient to maximize success.

## Conclusions

We selected, culturally adapted, and tested the acceptability of a behavioral intervention to improve retention in HIV care and adherence to ART medication in a rural Mozambican context. The adapted intervention was acceptable to clinicians, traditional healers, and potential clients. The process—piloting the intervention through theater presentations and using feedback to tailor our activities—allowed us to address potential barriers to implementation. If traditional healers in rural Mozambique can deliver this retention and adherence intervention, this may be a model for other regions of SSA where traditional healers are ubiquitous and interested in establishing a formal relationship with the health system. Something must be done to overcome the severe shortages in the health workforce, and engagement of healers as treatment partners is an option that merits further evaluation.

## Abbreviations

ADAPT-ITT: Assessment-Decision-Administration-Production-Topical Experts-Integration-Training-Testing
ART: Antiretroviral therapy
CDC: Centers for Disease Control and Prevention
PEPFAR: President’s emergency plan for AIDS relief
PLHIV: Persons living with HIV
sIMB-CIM: Situated-information motivation behavioral skills model of health care initiation and maintenance
SSA: Sub-Saharan Africa
